# Offspring of older parents are smaller—but no less bilaterally symmetrical—than offspring of younger parents in the aquatic plant *Lemna turionifera*

**DOI:** 10.1002/ece3.3697

**Published:** 2017-12-03

**Authors:** Eric J. Ankutowicz, Robert A. Laird

**Affiliations:** ^1^ Department of Biological Sciences University of Lethbridge Lethbridge AB Canada

**Keywords:** aging, Araceae, asymmetry, Lemnoideae, mirror image, shape analysis

## Abstract

Offspring quality decreases with parental age in many taxa, with offspring of older parents exhibiting reduced life span, reproductive capacity, and fitness, compared to offspring of younger parents. These “parental age effects,” whose consequences arise in the next generation, can be considered as manifestations of parental senescence, in addition to the more familiar age‐related declines in parent‐generation survival and reproduction. Parental age effects are important because they may have feedback effects on the evolution of demographic trajectories and longevity. In addition to altering the timing of offspring life‐history milestones, parental age effects can also have a negative impact on offspring size, with offspring of older parents being smaller than offspring of younger parents. Here, we consider the effects of advancing parental age on a different aspect of offspring morphology, body symmetry. In this study, we followed all 403 offspring of 30 parents of a bilaterally symmetrical, clonally reproducing aquatic plant species, *Lemna turionifera*, to test the hypothesis that successive offspring become less symmetrical as their parent ages, using the “Continuous Symmetry Measure” as an index. Although successive offspring of aging parents older than one week became smaller and smaller, we found scant evidence for any reduction in bilateral symmetry.

## 1 INTRODUCTION

Offspring quality decreases with parental age in many taxa (Kern, Ackermann, Stearns, & Kawecki, 2001; Priest, Mackowiak, & Promislow, 2002). Offspring of older parents may experience reduced life span (the “Lansing effect”: Lansing, 1947, reviewed in Gavrilov & Gavrilova, 1997; Priest et al., 2002; Lind, Berg, Alavioon, & Maklakov, 2015), reduced reproductive capacity (Wangermann & Ashby, 1950, 1951; Bowhuis, Charmantier, Verhulst, & Sheldon, 2010; Gillespie, Russell, & Lummaa, 2013), and, consequently, reduced fitness (Barks & Laird, 2015), compared to offspring of younger parents. The negative effect of parental age on offspring quality is a manifestation of senescence, in addition to the more familiar age‐related declines in the parents’ survival and reproduction. Such “parental age effects” are important because they can alter population growth and age structure, and may increase the rate at which the force of natural selection declines with advancing age, with subsequent feedback effects on the evolution of demographic trajectories and longevity (Barks, 2015; Barks & Laird, 2015).

In addition to their influence on the timing of offspring life history milestones, parental age effects can also help explain variation in offspring morphology. For example, in aquatic plants in the genus *Lemna* (“duckweed”), offspring of younger parents are larger, on average, than those of older parents (Wangermann & Ashby, 1950, 1951; Barks & Laird, 2015, 2016). Here, in addition to offspring size, we also consider the effects of advancing parental age on a different aspect of offspring morphology: body symmetry. Fluctuating asymmetry is the random, nondirectional departure from bilateral symmetry that occurs during many organisms’ development (Van Valen, 1962; see recent review by Graham, Raz, Hel‐Or, & Nevo, 2010). Such departures may reflect underlying developmental instability stemming from genetic effects (e.g., Tsujino & Takahashi, 2012) or environmental stress (e.g., Beasley, Bonisoli‐Alquati, & Mousseau, 2013). Just as older parents, when experiencing general age‐related physiological deterioration, may produce shorter‐lived, lower‐reproducing, and smaller offspring, so too can we hypothesize that they may be less able to produce bilaterally symmetrical offspring, compared to their younger counterparts. Supporting this hypothesis, Colines, Cabrera Rodríguez, Hasson, Carreira, and Frankel (2015) found strong evidence of progressively increasing wing asymmetry and leg asymmetry in *Drosophila melanogaster* of older versus younger parents (but also see more equivocal results in Parsons, 1962; Wakefield, Harris, & Markow, 1993).

In this study, we followed all 403 offspring of 30 parents of a bilaterally symmetrical aquatic plant species, in the aforementioned *Lemna* genus, to investigate parental age effects on offspring size and symmetry. Specifically, we tested the hypotheses that offspring size and bilateral symmetry decrease with advancing parental age. Although successive offspring of aging parents older than 1 week became smaller and smaller, we found no evidence of a reduction in bilateral symmetry.

## 2 MATERIALS AND METHODS

### 2.1 Study species

**Figure 1:**
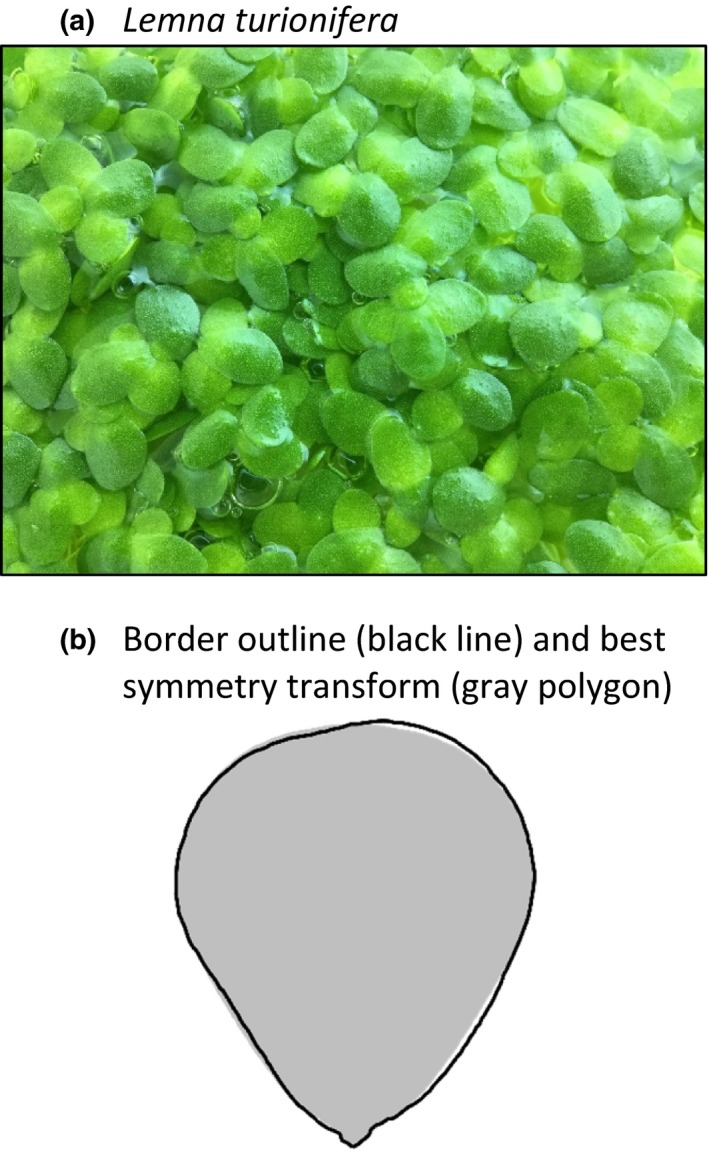
Images of *Lemna turionifera*. (a) *L. turionifera* is a small, fast‐growing, free‐floating, aquatic plant. (b) Example frond outline (black line) and best symmetry transform (closest bilaterally symmetrical shape according to the CSM criterion; gray polygon). This frond is highly bilaterally symmetrical, so its outline is similar to the best symmetry transform

*Lemna turionifera* Landolt (Araceae) is a small, floating, aquatic plant in the subfamily Lemnoideae. Physiological individuals in this genus have very simple morphology, being composed of a single “frond” of leaf and stem developmental origin, and a single root (Lemon & Posluszny, 2000). The frond is typically oval, with one axis of bilateral symmetry and one end pointier than the other (i.e., shaped somewhat like the outline of an egg or many types of guitar pick; Figure 1). Thus, *Lemna* fronds exhibit “object symmetry” (symmetry within an object) as opposed to “matching symmetry” (symmetry between two objects; Mardia, Bookstein, & Moreton, 2000; Klingenberg, Barluenga, & Meyer, 2002). Most reproduction in *Lemna* is via the asexual production of daughter fronds that detach from two meristematic pockets located on either side of the pointier end of the frond (Landolt, 1986). Under laboratory conditions favorable for survival and clonal reproduction, we have found that most individuals live between approximately 10 and 45 days, and produce approximately 5–20 daughters during this time (i.e., in closely related *Lemna minor*; Barks & Laird, 2015).

*Lemna* species have long been model systems for studying parental age effects (e.g., Ashby & Wangermann, 1949; Wangermann & Ashby, 1950). Frond surface area, total number of offspring produced, life span, and more direct measures of fitness have been found to be affected by the age of a focal frond's parent when the focal frond detached (Barks & Laird, 2015). Our anecdotal observations suggest that daughter fronds of older parents may be less bilaterally symmetrical compared to those of younger parents (a possible exception is that the very first daughter fronds produced by the meristematic pockets may be less symmetrical than the next several subsequent ones; see Figure S2 in Barks & Laird, 2016). Others have also noted deviations from typical daughter development in older parents (e.g., Lemon & Posluszny, 2000). Here, we formally investigate variation in offspring size and shape as a function of parental age. Unlike most plants, individual *L. turionifera* fronds have determinate growth, making them especially suitable for such a study.

### 2.2 Plant rearing

We used a strain of *Lemna* (“Wat A”) originally collected from a small wetland on the University of Lethbridge campus (49.6792°N, 112.8726°W). The axenic, single‐genotype stock culture was established according to methods described in Barks and Laird (2015). The strain was initially identified as *L. minor* (Barks, 2015 (ch. 3), Barks & Laird, 2016); however, the species identity was later determined by DNA barcoding to be *L. turionifera* (P. M. Barks, Z. W. Dempsey, T. M. Burg, and R. A. Laird, unpublished data; GenBank accession numbers MG000422 and MG000496). In the evitable trade‐off between the number of strains examined and the sample size per strain, we elected to strongly emphasize the latter (i.e., using a single strain and a large sample size) to better distinguish among candidate statistical models.

Upon removal from the stock culture, plants were grown individually under sterile conditions in 60 × 10 mm Petri dishes in 10 mL half‐strength Schenk and Hildebrandt growth medium (Sigma‐Aldrich, St. Louis, MO, USA) to which we added 6.7 g/L sucrose, 0.067 g/L yeast extract, and 0.34 g/L tryptone to aid in the detection of contamination. Plants were transferred to a new Petri dish with new growth medium every 4 days, unless fungal contamination was detected, in which case they were also transferred ahead of schedule. Focal plants were marked with a tiny speck of autoclaved diluted India ink.

Plants were grown on a four‐shelf unit, each shelf with a six‐bulb fixture (Agrobrite FLT46; Hydrofarm, Petaluma, CA, USA) with high‐output fluorescent grow bulbs (T5, 54 W, 6400K) positioned 25 cm above the plants. Plants grew on a 15:9 light:dark schedule. The average temperatures during the light phase varied by shelf (top shelf: 29.7°C, second shelf: 28.0°C, third shelf: 27.5°C, bottom shelf: 23.8°C). During the dark phase, the room temperature was 21.5°C.

### 2.3 Daily tracking

We started with 30 “parent” plants, positioned randomly among available positions on the four shelves. Each parent plant was the first daughter of a first daughter of a first daughter of the next daughter to detach from a plant after it was taken from the stock culture. (Descendants of multiple generations of first daughters were used to avoid the possibility of grandparental or other multigenerational effects; Barks & Laird, 2016.) We tracked the parents daily, recording the date that daughters detached, and removed these daughters to their own Petri dishes, which were also positioned randomly among available positions on the shelves. The daughters themselves (hereafter called “focal plants”; *n* = 403) were also marked with diluted India ink and tracked daily, with *their* daughters (i.e., the granddaughters of the initial parents) discarded as soon as they detached.

### 2.4 Image capture

Late in life, after its last daughter had detached, each focal plant was photographed with a microscope‐mounted digital camera. Plants were first removed from their growth medium, gently flattened between a microscope slide and cover, and then photographed. During this process, high contrast and sharply focused frond margins were emphasized, as this led to images that were optimal for shape analyses. Delicate fronds damaged during this process were excluded from main analyses (final *n* = 310 focal plants).

### 2.5 Size and shape: Continuous Symmetry Measure, area, perimeter, and circularity

Measures of symmetry typically compare homologous anatomical landmarks on corresponding left and right structures (e.g., on flies’ wings (Colines et al., 2015) and zygomorphic flowers (Gómez, Perfectti, & Camacho, 2006)) or on left and right sides of the same structure (Klingenberg et al., 2002). *Lemna turionifera* has few obvious and consistent anatomical landmarks on the edge of the fronds, other than perhaps the point farthest from the centroid of its outline. Thus, we instead used the Continuous Symmetry Measure of bilateral symmetry (CSM) developed by Zabrodsky, Peleg, and Avnir (1992), an index that uses arbitrary reference points on a shape's outline rather than fixed anatomical landmarks. Our method is similar to that described by Graham et al. (2015).

Before proceeding, it is important to note that “[within] the context of continuous symmetry measures (CSMs), shape asymmetry cannot be easily classified into fluctuating asymmetry, directional asymmetry, or antisymmetry” (Graham et al., 2015, p. 266). Thus, although concepts of fluctuating asymmetry motivated our hypothesis that offspring symmetry declines with parental age, we emphasize that we test for parental age‐related changes in bilateral symmetry in general, rather than changes in fluctuating asymmetry in particular. This is a constraint of studies of so‐called inconsistent objects that do not have homologous left and right landmarks (Graham et al., 2015).

Continuous Symmetry Measure operates on a polygon representing the outline of the shape in question (e.g., black line in Figure 1b). Obtaining a polygon outline from an image requires several initial processing steps:

First, the image is thresholded using Otsu's method (Otsu, 1979). Thresholding turns a gray scale or color image into a two‐class image, that is, a binary image where 1‐pixels represent the foreground (in this case the frond) and 0‐pixels represent the background. Here, thresholding was performed on the inverse of the blue channel of color (RGB) plant images (Graham et al., 2015). The intensity threshold for an image (i.e., the intensity cutoff between pixels assigned to the 0‐ and 1‐pixel classes) was determined as the value that maximized between‐class variance (Otsu, 1979).
The second step is the detection of connected components in the binary image (using 4‐connectivity, in the sense that pixels above, below, left, and right of a focal pixel are considered to be “connected” to it). Once the connected components are detected, all but the largest connected component (the frond) are deleted. This has the effect of filtering (or “cleaning up”) the image and removing any specks that survived thresholding.
Third, any holes in the image of the frond are detected and filled using a flood‐fill procedure.
Fourth, the boundary pixels are detected (a boundary pixel is a 1‐pixel adjacent to at least one 0‐pixel) and then ordered using Pavlidis’ algorithm (Pavlidis, 1982).
Fifth, equally spaced reference points are placed around the boundary. For all of our analyses described here, we used *L* = 200 reference points per image (as recommended by Graham et al., 2015).
Finally, the coordinates of the ordered reference points are normalized so that the greatest distance from the centroid to any of the reference points was one. (This is performed to make CSM scale‐independent.) The normalized polygon is then fed into the CSM‐calculating procedure.

The CSM procedure itself works as follows (Zabrodsky et al., 1992):

First, the reference points are paired by starting at an arbitrary point along the boundary of the normalized polygon and grouping together the next clockwise and counterclockwise reference points that have not been previously paired, until all *L* reference points are paired. There is the same number of unique pairings as there are reference points. When *L* is even, as it is here, half of these pairings occur when the arbitrary starting point is between reference points, in which case there are *L*/2 pairs. The other half of the pairings occur when the arbitrary starting point is itself a reference point; in this case, there are *L*/2 + 1 pairs as two reference points (the starting point and the reference point *L*/2 reference points away from it) are paired with themselves.
Second, the optimal reflection axis for the particular pairing is determined. The optimal reflection axis is the one that minimizes the averaged squared distance between the observed reference points and an equal number of reference points from a hypothetical bilaterally symmetrical shape (known as the “symmetry transform”; Figure 1b). The axis can be found analytically, as described in Zabrodsky et al. (1992).
Third, one member of each pair is reflected across the optimal axis and a new point is obtained by averaging the reflected point's coordinates with those of its pair‐mate. Another new point is obtained by reflecting the first new point back across the optimal axis. When this procedure is repeated for all pairs, the set of new points represents the points of the closest bilaterally symmetrical polygon to the observed polygon, according to the minimized average squared distance criterion, and given the current pairing of reference points.
Fourth, the average squared distance between every reference point and its counterpart in the closest bilaterally symmetrical polygon is calculated. CSM is the minimum such value over all possible pairings of reference points allowed by the pairing procedure discussed above (i.e., comparing the actual shape and the best symmetry transform). CSM values are nondirectional with a minimum value of zero (i.e., when the shape is perfectly bilaterally symmetrical).

In addition to this typical implementation of CSM, we also calculated another, related index, which we refer to as CSM_forced_. In this case, rather than minimizing the squared distance between every reference point and its counterpart in the closest bilaterally symmetrical polygon over all possible pairings of reference points, CSM_forced_ forces the pairing of points through the reference point with the greatest Euclidian distance from the shape's centroid. This has the effect of testing for bilateral symmetry through the pointy part of the frond, that is, through the putative axis of symmetry in *Lemna*. Often, especially in highly symmetrical fronds, CSM and CSM_forced_ are same or very similar, because the best axis of bilateral symmetry *is* the one that goes through or near the point farthest from the centroid. However, in some cases there was a discrepancy (especially in more asymmetrical fronds), which is why we calculated both.

Along with measures of symmetry, the (pre‐normalized) reference points were also used to estimate frond area and perimeter, after calibrating image pixels with absolute distance. Together, area and perimeter allowed for the calculation of circularity = 4π (area/perimeter^2^), an index with a maximum of 1 (i.e., a perfect circle) and a minimum approaching 0 (i.e., a highly convoluted shape).

All the image analysis steps were evaluated in MATLAB (version R2016a, The MathWorks Inc., Natick, USA, 2016). Code is archived in Dryad (Ankutowicz & Laird, 2017).

### 2.6 Statistical analyses: Frond size and shape

Statistical analyses were performed in R (version 3.3.3., R Foundation for Statistical Computing, Vienna, Austria, 2017). Code and data are archived in Dryad (Ankutowicz & Laird, 2017).

The analyses for frond size and shape were based on the analyses of Barks and Laird (2015), which themselves followed Zuur, Ieno, Walker, Saveliev, and Smith (2009).

The goal was to test the effects of parental age on offspring size (frond surface area and perimeter) and shape (circularity, CSM, and CSM_forced_), while accounting for shelf effects and the potential nonindependence of offspring from the same parent. Because senescence is often a nonlinear phenomenon, we tested polynomials up to the cubic term.

First, we fit linear mixed models for each of the following random‐effects structures: (1) random intercept and slope terms for parent identity, (2) random intercept term for parent identity, and (3) no random effects. These models were fit with restricted maximum likelihood using the *nlme* package and the functions *lme* or *gls* (i.e., for models with and without random effects, respectively; Pinheiro, Bates, DebRoy, & Sarkar, 2017). We determined best random‐effects structures as those that minimized AIC_C_ among nine candidates (three random‐effects structures for each of linear, quadratic, and cubic models). The three shape metrics had consistent best random‐effects structures across the three polynomials (i.e., “no random effects”). However, this was not so for the size metrics: In the case of area, “random intercept for parent identity” yielded the lowest AIC_C_ for the linear model, whereas “random intercept and slope terms for parent identity” yielded the lowest AIC_C_ for the quadratic and cubic models. Likewise, in the case of perimeter, “no random effects” had the lowest AIC_C_ for the linear model, whereas “random intercept and slope terms for parent identity” had the lowest AIC_C_ for the quadratic and cubic models. In both cases, the ΔAIC_C_ values comparing best‐ and second‐best‐fitting random‐effects structures were greater when the “random intercept and slope terms for parent identity” was best‐fitting than when a different random‐effects structure was best‐fitting. Further, the best fixed‐effects models for area and perimeter were ultimately found to involve cubic functions of parental age (see below), for which the most highly specified random‐effects structures were best‐fitting. For these reasons, for area and perimeter, we used the “random intercept and slope terms for parental identity.”
Second, we fit all eight possible fixed‐effects models (i.e., up to the zeroth‐, first‐, second‐, and third‐degree polynomials of parental age crossed with the presence/absence of shelf), using *lme* or *gls* to describe the full model and *dredge* (in the MuMIn package; Bartoń, 2017) to investigate the subsets. Once again, the best model was deemed to be the one with the lowest AIC_C_. In two cases (perimeter and circularity, the latter transformed as described below), shelf was included in the best‐fit model. Because the effects of shelf were small, and because shelf was a “nuisance variable” and not of interest in and of itself, in the main text we report the predicted values averaged across levels of shelf. In the supplementary material, we also report the corresponding models that include shelf (Figure S1 in Appendix S1).
Third, we assessed homoscedasticity and normality visually using histograms of residuals, quantile–quantile plots, and scatter plots of residuals versus predicted values. CSM and CSM_forced_ were right‐skewed; to satisfy model assumptions, these variables were ln(*x*) transformed and reanalyzed as above. Circularity was left‐skewed; to satisfy model assumptions, this variable was ln(1 – *x*) transformed and reanalyzed as above. Note that the “1 – *x*” operation, applied to convert the observed skewness rightward so that the further ln transformation would be effective, necessitates a reinterpretation of circularity wherein low values (1 − *x* ≈ 0) correspond to broadly circular shapes and high values (1 – *x* ≈ 1) correspond to convoluted shapes.

### 2.7 Statistical analyses: Frond inclusion/exclusion

A number of fronds were damaged during the data collection process; we sought to determine whether the probability of exclusion was related to parental age:

Thus, we fit generalized linear mixed models (binomial distribution for binary data—fronds were either “included” or “excluded”) for each of the following random‐effects structures: (1) random intercept and slope terms for parent identity, (2) random intercept term for parent identity, and (3) no random effects. We used the functions *glmer* and *glm* for models with and without random effects, respectively (the former being in the package lme4; Butes, Maechler, Bolker, & Walker, 2017). The model with the lowest AIC_C_ was chosen as the best‐fit model.

## 3 RESULTS

### 3.1 Size: Area and perimeter

Frond surface area and perimeter were both best‐fit by cubic models of parental age and included random intercept and slope terms for parental identity. Shelf was excluded from the best‐fit model for area, but included for perimeter. However, in the case of perimeter, ΔAIC_C_ < 2 for a cubic model that excluded the shelf term, indicating that this second model fit approximately as well as the putative best‐fit model (Table 1).

**Table 1:** Top five models for two measures of offspring size (area and perimeter) and three measures of shape (circularity, CSM, and CSM_forced_) as functions of parental age (*P*) and shelf (*S*). The best‐fit models for each measure of offspring shape were the ones with lowest AIC_C_ (in bold)

Dependent variable	Model^a^	*df*	log likelihood	AIC_C_	ΔAIC_C_	AIC_C_ weight
Area^b^	***P*** ^**3**^	**8**	–**348.7**	**713.8**	**0.00**	**0.937**
	*P* ^3^ + *S*	11	–348.1	719.2	5.38	0.063
	*P* ^2^	7	–371.3	756.9	43.15	0.000
	*P* ^2^ + *S*	10	–370.9	762.6	48.79	0.000
	*P*	6	–435.5	883.3	169.46	0.000
Perimeter^b^	***P*** ^**3**^ + ***S***	**11**	–**502.4**	**1027.8**	**0.00**	**0.503**
	*P* ^3^	8	–505.8	1028.1	0.37	0.417
	*P* ^2^ + *S*	10	–506.0	1032.7	4.90	0.043
	*P* ^2^	7	–509.3	1033.0	5.27	0.036
	*P* + *S*	9	–534.3	1087.1	59.34	0.000
ln(1 – circularity)^c^	***P*** + ***S***	**6**	**235.0**	–**457.7**	**0.00**	**0.292**
	*P* ^2^ + *S*	7	235.8	–457.2	0.47	0.231
	*S*	5	233.5	–456.5	1.18	0.162
	*P*	3	230.7	–455.3	2.34	0.091
	*P* ^3^ + *S*	8	235.8	–455.2	2.53	0.082
ln(CSM)^c^	***P*** ^**3**^	**5**	–**441.4**	**893.0**	**0.00**	**0.594**
	*P* ^3^ + *S*	8	–439.5	895.4	2.40	0.179
	*P*	3	–445.5	897.0	4.03	0.079
	*P* ^2^	4	–444.7	897.5	4.45	0.064
	(intercept‐only)	2	–447.2	898.5	5.48	0.038
ln(CSM_forced_)^c^	**(intercept‐only)**	**2**	–**476.7**	**957.5**	**0.00**	**0.394**
	*P*	3	–476.0	958.1	0.59	0.293
	*P* ^2^	4	–476.0	960.1	2.62	0.106
	*P* ^3^	5	–475.1	960.4	2.93	0.091
	*S*	5	–475.7	961.6	4.08	0.051

a Superscripts accompanying parental age (*P*) denote maximum polynomial degree of fitted model.

b Models include random intercept and slope terms for parent identity.

c Models do not include random effects.

**Figure 2:**
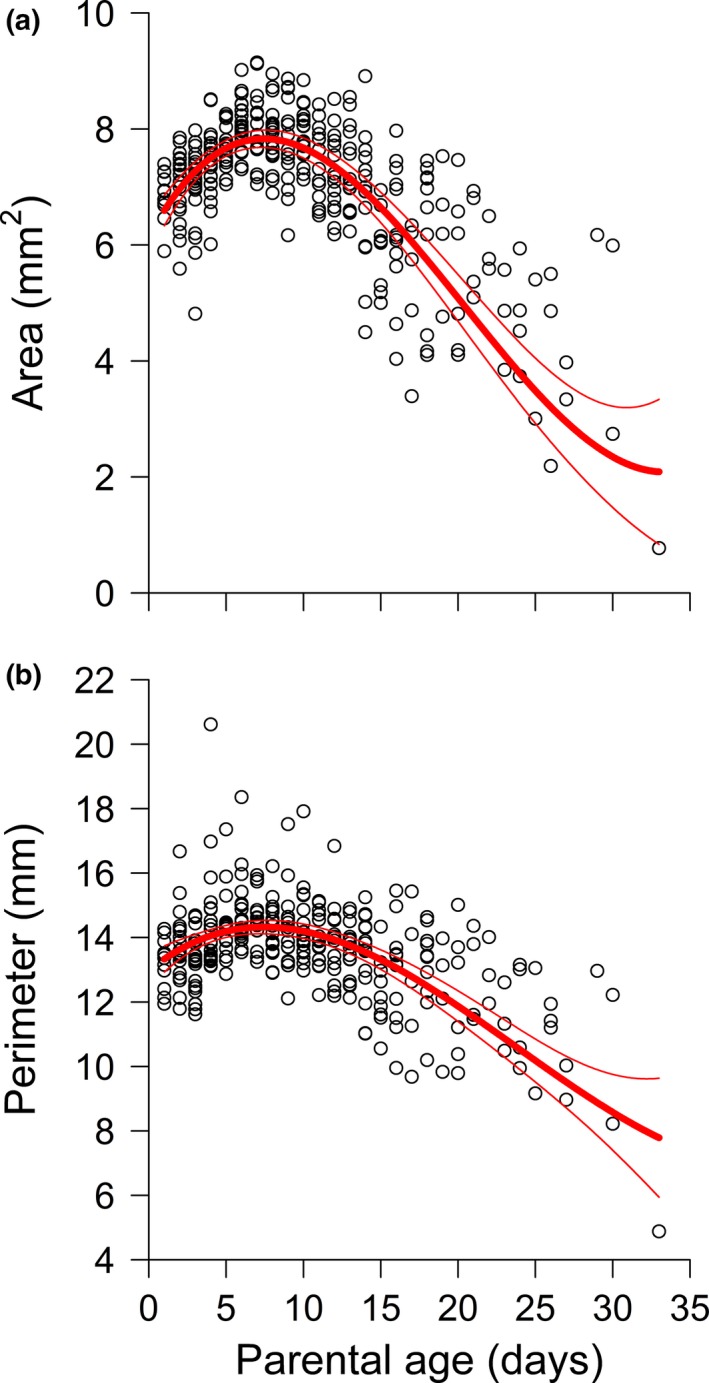
Two measures of offspring size as functions of parental age. (a) Frond surface area and (b) frond perimeter. Best‐fit models are given in Table 1. Predictions (thick lines) are provided with 95% confidence bands (thin lines). Sample size: *n* = 310

In very young parents, average offspring surface area and perimeter increased with parental age, reaching peaks in parents of approximately 1 week in age. Thereafter, average offspring size dropped precipitously with parental age and was the lowest in the oldest parents (Figure 2).

### 3.2 Shape: Circularity, CSM, and CSM_forced_

Circularity (ln(1 − *x*) transformed) was best‐fit by a linear model of parental age that included a shelf term. CSM (ln(*x*) transformed) was best‐fit by a cubic model of parental age that did not include a shelf term. CSM_forced_ (ln(*x*) transformed) was best‐fit by an “intercept‐only” model (no effect of parental age) that did not include a shelf term. In all three cases, the models did not include any random terms for parental identity. In the case of circularity and CSM_forced_, at least one alternative model had ΔAIC_C_ < 2 (Table 1).

**Figure 3:**
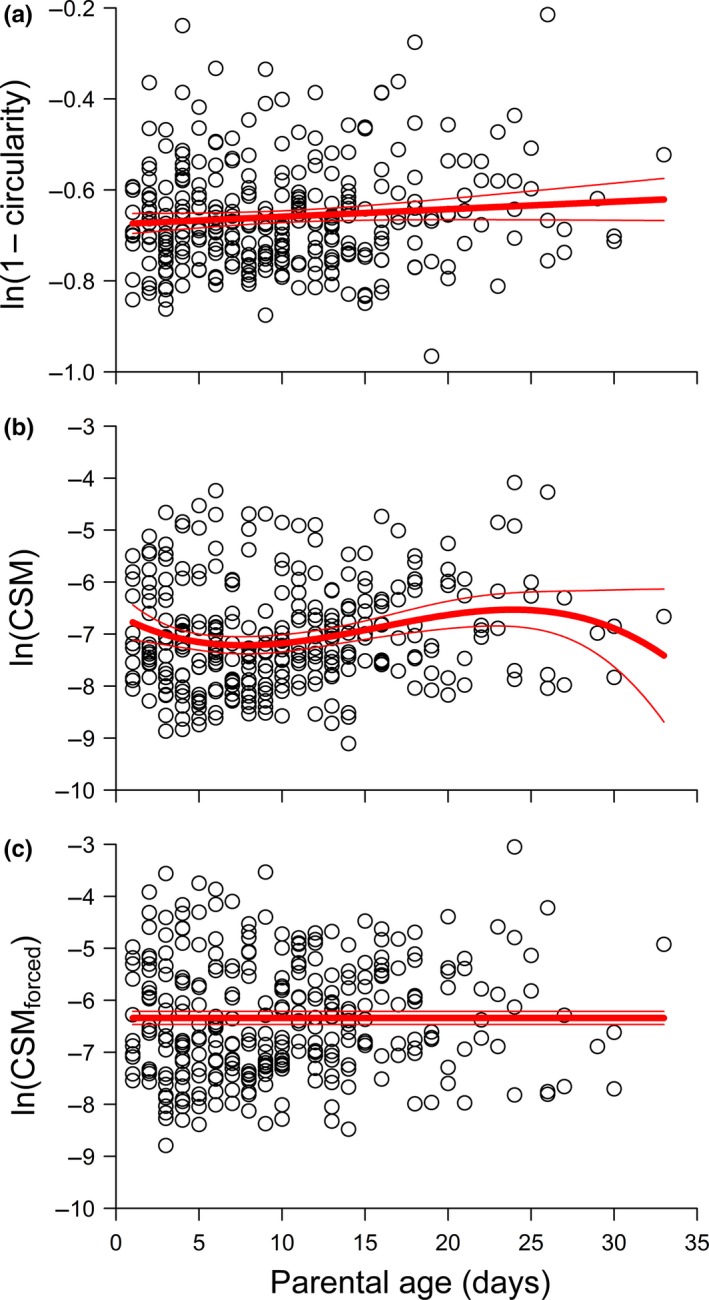
Three measures of offspring shape as functions of parental age. (a) ln(1 – circularity), (b) ln(CSM), (c) ln(CSM_forced_). In panel (a), lower values of ln(1 – circularity) correspond to more circular fronds. In panels (b) and (c), lower values of ln(CSM) and ln(CSM_forced_) correspond to more bilaterally symmetrical fronds. Best‐fit models are given in Table 1. Predictions (thick lines) are provided with 95% confidence bands (thin lines). Sample size: *n* = 310

In contrast to offspring size, relationships between offspring shape and parental age were equivocal across competing models and/or weak (Table 1; Figure 3). While the best‐fit model for ln(1 – circularity) indicated that offspring of older parents were less circular than offspring of younger parents (Figure 3a), the fit was noisy and this model was only marginally better fitting than a model that did not include any parental age effects at all (i.e., ΔAIC_C_ = 1.18 for the shelf‐only model; Table 1). For ln(CSM), the best‐fitting cubic model of parental age did not show any directional trends with offspring of 1‐day‐old, middle age (~18‐day‐old), and elderly (~30‐day‐old) parents being approximately equally bilaterally symmetrical (Figure 3b). For ln(CSM_forced_), the shape index that most closely reflects the putative axis of bilateral symmetry in *Lemna*, the best‐fit model did not include parental age (Figure 3c).

### 3.3 Frond inclusion/exclusion

**Figure 4:**
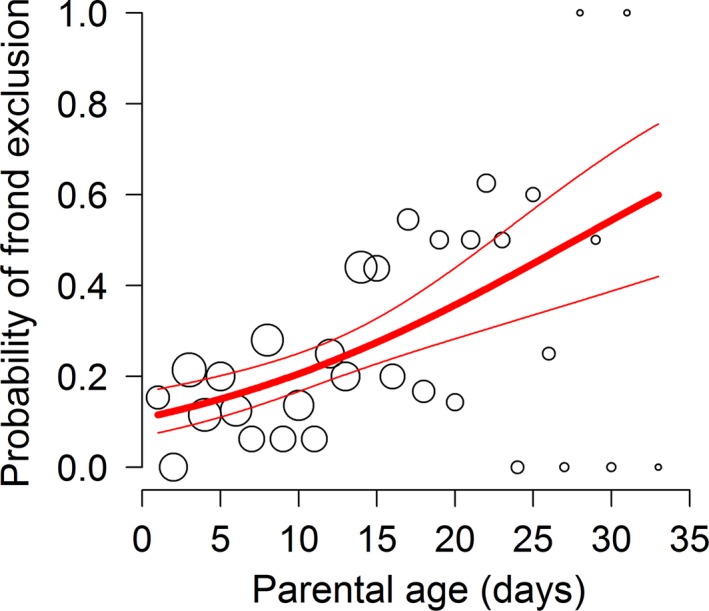
Probability of frond exclusion as a function of parental age. The best‐fit model was a generalized linear model with binomial distribution and no random effects (i.e., logistic regression; AIC_C_ = 419.2, *z* = 4.425, *p* = 9.65 × 10^−6^). Prediction (thick line) is provided with 95% confidence bands (thin lines). Symbol area is proportional to sample size for a given parental age. Total sample size: *n* = 403; number of fronds included: *n* = 310

Logistic regression (with no random effects associated with parent identity) demonstrated that the predicted probability of frond exclusion was positively related to parental age (Figure 4).

## 4 DISCUSSION

Successive offspring became smaller, on average, as their parents aged (i.e., in parents older than 1 week; Figure 2), strongly supporting previous studies that report a decrease in offspring size with parental age in *Lemna* (Wangermann & Ashby, 1950, 1951; Barks & Laird, 2015, 2016). An interesting complication to this general trend is that average offspring size actually *increased*, albeit slightly, over the first week of parents’ lives (Figure 2). This phenomenon has also been noted by us (Barks & Laird, 2016) and others (Claus, 1972), but not in other studies (Wangermann & Ashby, 1951; Barks & Laird, 2015). In *Lemna*, offspring of young parents often start to develop, while those parents are still attached to *their* parents, and are still growing (Lemon & Posluszny, 2000). This may prevent offspring of young parents from attaining the size of offspring that detach from full‐size, mature parents (Barks & Laird, 2016). However, the fact that in some studies offspring size is a monotonically decreasing function of parental age suggests that there are further complications at play (Wangermann & Ashby, 1951; Barks & Laird, 2015). Future research should consider the hypothesis that, across *Lemna* strains, maximum offspring size is positively related to the parent's age when it detaches from *its* parent. In any case, the early‐life increase in offspring size with parental age was brief in duration, and small in magnitude, compared to the long, large decrease in offspring size in older parents (Figure 2).

Regarding offspring shape, we found scant evidence that fronds of older parents were less bilaterally symmetrical than their earlier‐detached sister fronds (Figure 3b,c), thus failing to support our prediction, and in contrast to the results on wing asymmetry in *Drosophila* by Colines et al. (2015). We propose that a negative relationship between offspring bilateral symmetry and parental age may be more common in species for which bilateral symmetry is closely related to fitness. For example, it may be that departures from bilateral symmetry in the wings of flying animals may have important aerodynamic effects, affecting flight ability and, in turn, fitness (e.g., Thomas, 1993; but see Johnson & Cartar, 2014). As another example, floral bilateral symmetry may be adaptive in some plant species in which it has been shown to be associated with increased pollinator visitation and fitness (Gómez et al., 2006). On the other hand, although *L. turionifera* is a bilaterally symmetrical species, we see no obvious reasons why strict bilateral symmetry should be advantageous. If it turns out there is no selection to maintain perfect bilateral symmetry in offspring, it is perhaps not surprising that bilateral symmetry does not show obvious signs of deterioration with parental age, compared to other, fitness‐critical traits such as latency to first reproduction, number of grand‐offspring, size, and intrinsic rate of increase (Barks & Laird, 2015).

During data collection, it was necessary to flatten fronds to avoid any curling, which would obscure measurements of area, perimeter, and symmetry. While fronds were handled gently, this flattening process nevertheless led to almost one‐quarter of the original 403 fronds being excluded due to damage they incurred. Interestingly, the probability that a frond was damaged and excluded increased with the age of that frond's parent (Figure 4). This could represent another unanticipated kind of adverse parental age effect; that is, offspring of older parents are more damage‐prone than offspring of younger parents. However, it is important to note that this was not an a priori prediction, and it deserves closer scrutiny and purpose‐designed experiments (e.g., to consider alternatives such as the hypotheses that curled fronds require more pressure to flatten (increasing their damage risk), and curling increases with parental age).

Perhaps a more important issue is whether the increase in frond exclusion with parental age could have influenced the results with respect to offspring size and shape. We argue that this is unlikely to be important. In terms of area and perimeter, if such an influence were present (e.g., if the “extra” excluded offspring of older parents were disproportionately large individuals), it still does not explain why the *minimum* size of offspring was much lower for older parents (Figure 2). In addition, fortuitously, we note that as a matter of protocol we still collected data from most of the fronds that were damaged (and therefore excluded from the main analyses). Torn fronds’ areas would still be accurate (in the same sense that the area of a torn, gently flattened orange peel, is an accurate estimate of the orange's surface area prior to peeling), even though all the other metrics would not. When all of these fronds were included (*n* = 384), the relationship between area and parental age was much the same as in Figure 2a, while the probability that a frond was *still* excluded (i.e., because it disintegrated or was otherwise too damaged to be photographed at all) increased much more shallowly with parental age compared to Figure 4. (And, in any case, this relationship is less important in this expanded data set since such a smaller total proportion of fronds were excluded.) The results of these additional analyses are provided in the supplemental information (Figure S2 and S3 in Appendix S1).

No such supplemental analysis is possible for offspring shape; however, the notion that a real change in offspring shape was masked by more symmetrical offspring being differentially prone to damage, and even then only when they had older parents, is, we argue, less parsimonious than the null explanation that there was no such relationship in the first place. We advocate that future research into parental age effects should examine the probability of sample exclusion with parental age (as we have in Figure 4) and should make every effort to minimize sample loss at every parental age.

In this study, we used a single *L. turionifera* strain so that we could attain a sufficient sample size to distinguish among candidate statistical models. The need for a large sample is particularly acute in studies involving aging, as the number of individuals attenuates in old age classes. However, future work should consider how genetic diversity affects parental age effects on offspring morphology.

Parental age effects appear to be common in *Lemna* species. However, it is clear that some offspring traits (e.g., size, correlates of fitness) exhibit much stronger parental age effects than others (e.g., shape). An important task will be to develop a firmer theoretical foundation for parental age effects that helps predict situations in which we should expect them to occur and situations in which we should not.

## CONFLICT OF INTEREST

None declared.

## AUTHOR CONTRIBUTIONS

EJA collected the data and wrote an initial draft. RAL designed the study, wrote or modified the analysis code, and wrote the final draft. Both authors commented on and approved the final draft.
