# Supplementary material for: Offspring of older parents are smaller—but no less bilaterally symmetrical—than offspring of younger parents in the aquatic plant Lemna turionifera
Source: Ecol Evol. 2017 Dec 3;8(1):679–87. doi: 10.1002/ece3.3697 (PMC5756881; doi:10.1002/ece3.3697)
Supplement: Supplementary file 1 [file ECE3-8-679-s001.pdf]

SUPPLEMENTARY MATERIAL: APPENDIX S1

Offspring of older parents are smaller—but no less bilaterally symmetrical—than offspring of younger parents in the aquatic plant *Lemna turionifera*

Eric J. Ankutowicz and Robert A. Laird*

Department of Biological Sciences, University of Lethbridge, Lethbridge, AB T1K 3M4, Canada

* Correspondence: robert.laird@uleth.ca

Figure S1 shows the model fits—including the effect of shelf—for frond perimeter and circularity (the latter ln(1 – x) transformed) versus parent age.

Figure S2 shows a comparison between the model fit for frond area versus parental age when all damaged fronds were excluded, compared to when those that were damaged, but nevertheless had area measurements taken, were included (i.e., only those with no area measurement were excluded).

Figure S3 shows a comparison between the probability of frond exclusion versus parental age when all damaged fronds were excluded compared to when only those that had no area measurement were excluded.

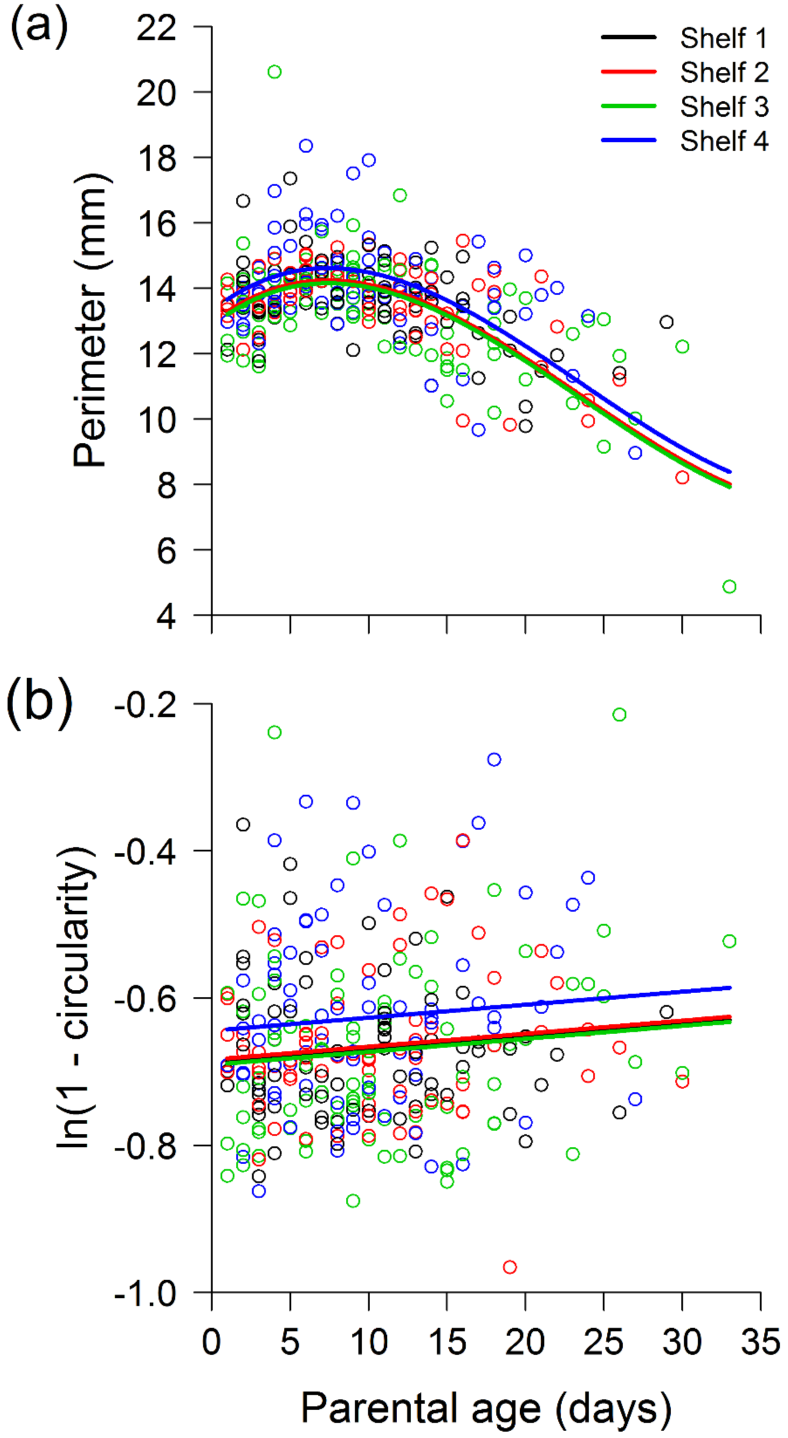

Figure S1. (a) Perimeter versus parental age, and (b) ln(1 – circularity) versus parental age; lower values of ln(1 – circularity) correspond to more circular fronds. Best-fit models are given in Table 1. Points for (a) and (b) are the same as in Fig. 2b and 3a, respectively; however, here predictions (thick lines) are given for the four different shelves in the plant stand. Point and line colors correspond to different shelf identities. Sample size: n = 310.

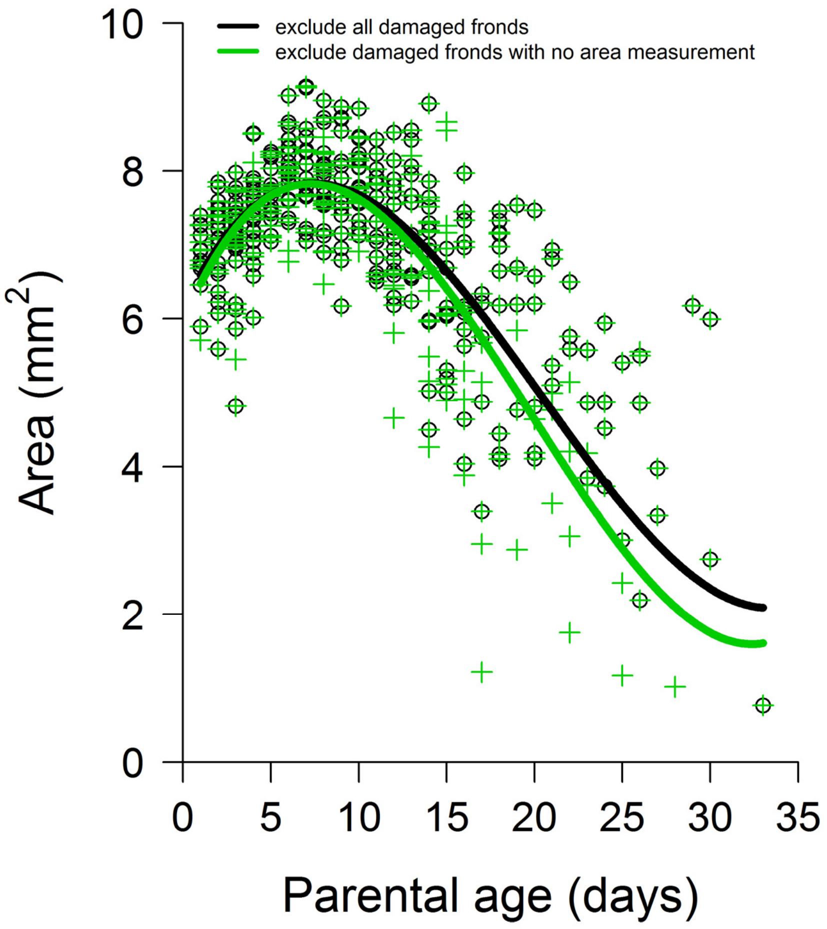

Figure S2. Frond surface area versus parental age when all damaged fronds are excluded (black lines and circles; equivalent to Fig. 2a; n = 310) or when only fronds with no area measurement are excluded (green lines and crosses; n = 384). The best-fit model for the former was a cubic model that did not include a Shelf term and included random intercept and slope terms for parent identity (Table 1). The equivalent model for the latter: df = 8, log likelihood = -492.3, AIC_C = 1001.0.

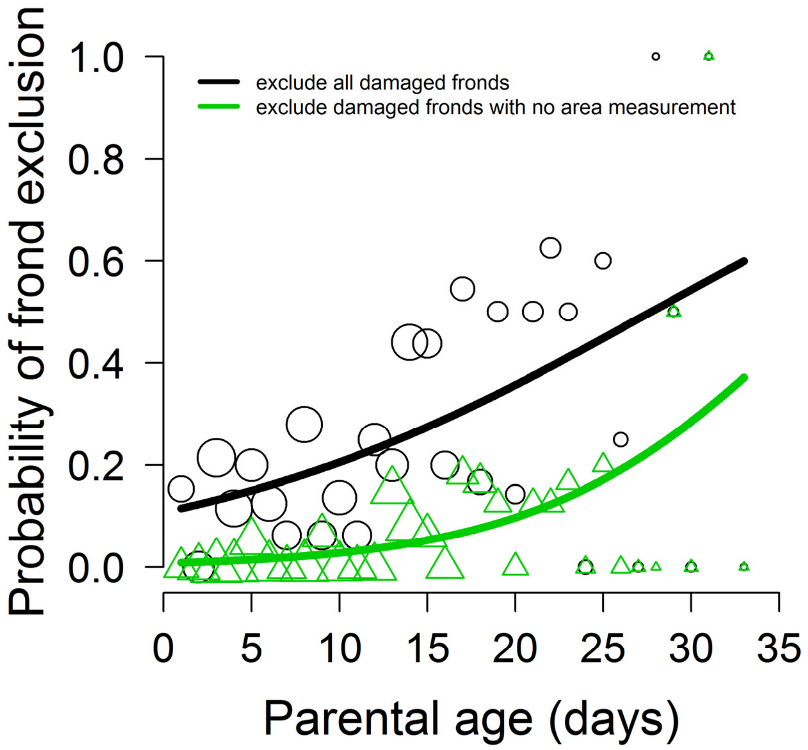

Figure S3. Probability of frond exclusion as a function of parental age when all damaged fronds are excluded (black lines and circles; equivalent to Fig. 4; $n_{included}$ = 310) or when only fronds with no area measurement are excluded (green lines and triangles; $n_{included}$ = 384). The best-fit model for the former was a generalized linear model with binomial distribution and no random effects (i.e., logistic regression; z = 4.425, $p = 9.65 \times 10^{-6}$). The equivalent generalized linear model for the latter: z = 4.023, $p = 5.76 \times 10^{-5}$. Symbol area is proportional to sample size for a given parental age. Total sample size: n = 403.
